# Infratentorial benign cystic meningioma mimicking a hemangioblastoma radiologically and a pilocytic astrocytoma intraoperatively: a case report

**DOI:** 10.1186/1752-1947-7-87

**Published:** 2013-03-28

**Authors:** Tan Kheng Guan, Devaraj Pancharatnam, Hari Chandran, Teoh Kean Hooi, Gnana Kumar, Dharmendra Ganesan

**Affiliations:** 1 Division of Neurosurgery, Department of Surgery, Faculty of Medicine, University of Malaya, Kuala Lumpur 50603, Malaysia; 2 Department of Biomedical Imaging, University Malaya Medical Centre, Kuala Lumpur, Malaysia; 3 Department of Pathology, University Malaya Medical Centre, Kuala Lumpur, Malaysia

**Keywords:** Benign infratentorial cystic meningioma, Hemangioblastoma, Pilocytic astrocytoma

## Abstract

**Introduction:**

Cystic meningiomas are rare variants of meningiomas; they can pose a radiological diagnostic dilemma.

**Case presentation:**

We present a rare case of a 30-year-old Chinese woman with a histopathological diagnosis of infratentorial cystic meningioma (World Health Organization Grade 1) in which the features in imaging modalities were suggestive of a hemangioblastoma. Intraoperatively, however, the gross macroscopic features were more in keeping with a pilocytic astrocytoma.

**Conclusion:**

In benign cystic meningiomas, particularly the infratentorial variety, radiological findings utilizing the various imaging modalities and intraoperative impressions may not be reflective of or in keeping with the final histopathological diagnosis.

## Introduction

Meningiomas are the most common primary non-glial intracranial tumors [1]. They are commonly described as a solid tumor and account for between 10% and 20% of all intracranial tumors in adults. The incidence of meningiomas rises with advancing age and is more common in females. However, cystic meningiomas are uncommon and the incidence varies from 1.6% to 10.0% of all meningiomas. By contrast, cystic meningiomas are more common in males [2]. Cystic meningiomas can pose a diagnostic dilemma radiologically. They can suggest other intracranial cystic masses: hemangioblastoma, astrocytoma, neuroblastoma or a metastatic tumor with a cystic or necrotic component [3]. We present a case of an infratentorial cystic meningioma mimicking a hemangioblastoma radiologically and pilocytic astrocytoma intraoperatively.

## Case presentation

A 30-year-old Chinese woman with a childhood history of acute lymphoblastic leukemia, currently in remission, presented to our Neurology clinic with a 3-week history of worsening headaches, giddiness and vomiting. The neurological examination revealed positive cerebellar signs in keeping with an infratentorial space-occupying lesion.

**Figure 1:**
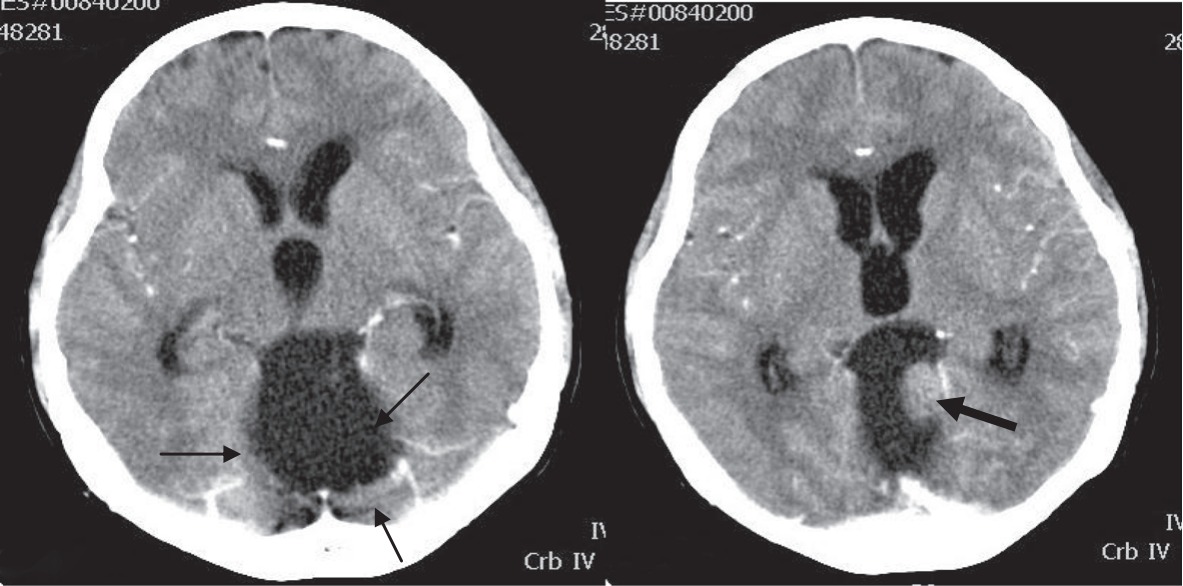
Axial contrast-enhanced computed tomography brain scan showing a large posterior fossa cystic mass (thin black arrows) with enhancing nodule (thick black arrow) compressing 4th ventricle causing obstructive hydrocephalus.

A computed tomography (CT) head scan revealed a large (4.0cm×4.1cm×4.8cm) posterior fossa cystic mass with an eccentrically located enhancing nodule measuring 1.5cm×1.9cm (Figure 1). This tumor was compressing the midbrain and 4th ventricle causing dilatation of the 3rd and lateral ventricles. There was compression of the cerebellum posteriorly resulting in tonsillar herniation. The initial radiological differential diagnosis of hemangioblastoma or pilocytic astrocytoma was based on the tumor’s characteristic appearance and location.

**Figure 2:**
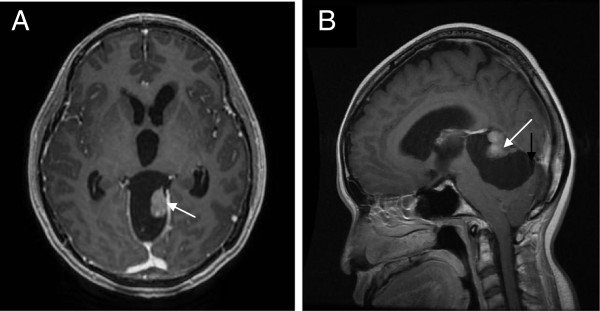
**Contrast-enhanced T1 axial (A) and sagittal (B) magnetic resonance imaging demonstrating the extra-axial posterior fossa cystic lesion from the posterior interhemispheric fissure (black arrow) with an enhancing mural nodule (white arrow).** No evidence of a dural tail was seen. Also noted were dilated 3rd and lateral ventricles.

**Figure 3:**
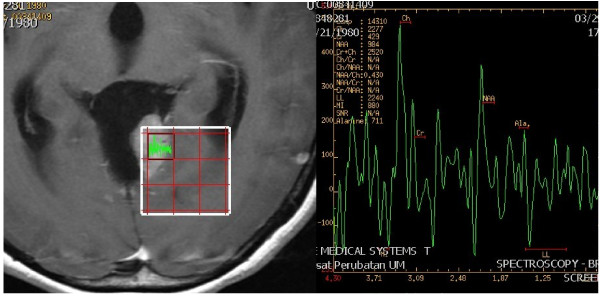
Magnetic resonance spectroscopy of the enhancing mural nodule from the posterior fossa cystic lesion showed reduced N-acetylaspartate level with increased choline-to-creatine ratio.

Subsequent magnetic resonance imaging (MRI) confirmed the cystic mass in the posterior fossa with an eccentrically located nodule lying adjacent to the vein of Galen. This nodule was hyperintense on T2-weighted and fluid-attenuated inversion recovery (FLAIR) sequences, hypointense on T1 weighted and showed avid enhancement with gadolinium (Figure 2). There was no evidence of a ‘dural tail’ enhancement. Findings of periventricular hyperdensities on T2 FLAIR in keeping with cerebrospinal fluid were indicative of acute hydrocephalus. A magnetic resonance spectroscopy (MRS) of the enhancing nodule showed non-specific reversal of the choline-to-creatine ratio with slight reduction in the N-acetyl acetate peak. Diffusion-weighted imaging and apparent diffusion coefficient (Figure 3) also showed restricted diffusion of the ‘mural nodule’. After reviewing the MRI findings, a radiological diagnosis of ‘hemangioblastoma’ was made. The patient was then scheduled for surgery.

The tumor was approached via the infratentorial supracerebellar corridor with the patient in a prone position. Frameless neuronavigation using a fine-cut (1.2mm) contrast-enhanced CT brain scan was utilized in planning the surgical access. Gross total excision of the tumor was achieved under microscope assistance. The cystic component of the lesion contained xanthochromic fluid. The well-circumscribed solid component was a greyish, soft vascular lesion attached to the undersurface of the tentorium cerebelli. The wall of the cyst was smooth with no nodular lesion, suggestive of a pilocytic astrocytoma. The wall was not excised. The deep venous complex was visualized and preserved. A blood transfusion was not needed. The symptomatology improved post-operatively. She was discharged on the 4th post-operative day.

**Figure 4:**
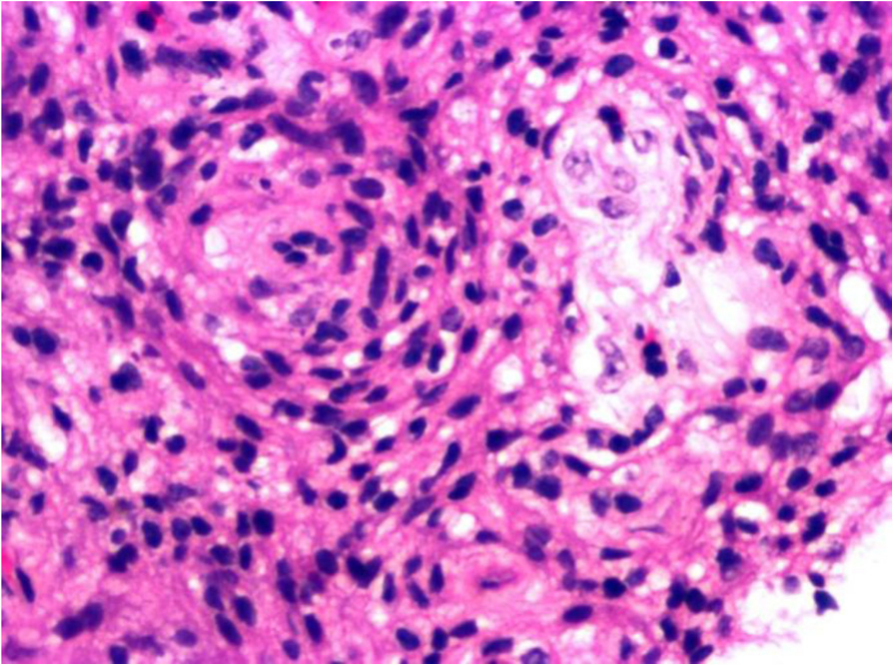
**Benign meningioma [World Health Organization Grade 1]: whorls of cells with oval nuclei seen in a syncytial-like background.** No significant mitotic activity or necrosis is evident.

**Figure 5:**
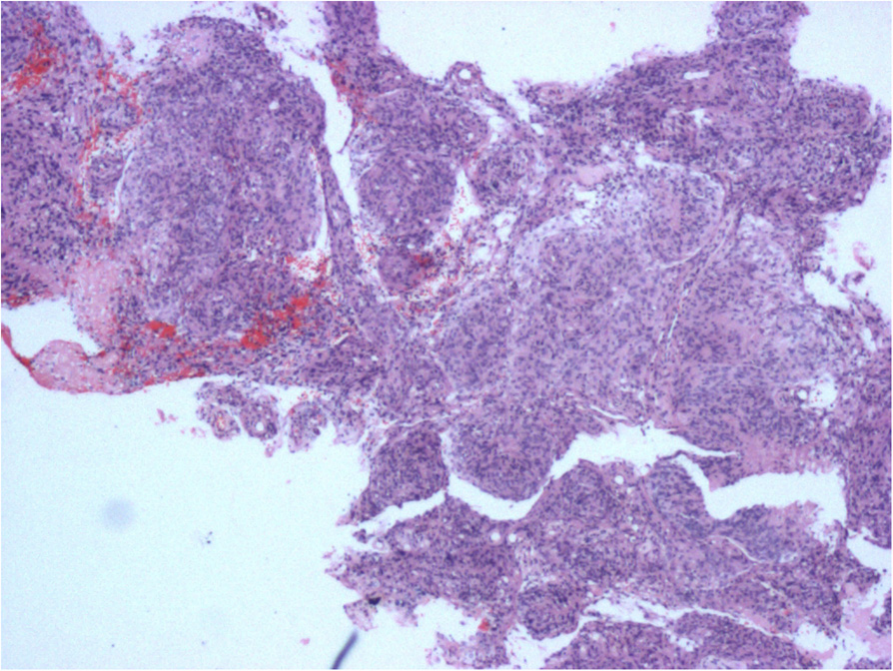
**Benign meningioma [WHO Grade 1] in which swirling whorls of cells with oval nuclei are seen in a syncytial like background.** Hematoxylin and eosin stain; magnification, ×200.

**Figure 6:**
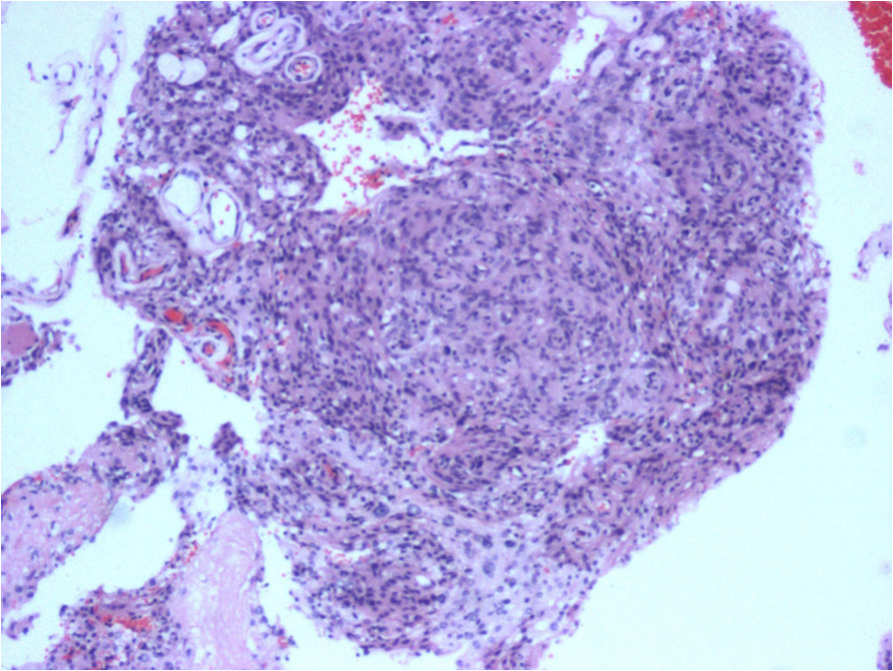
**Whorls of meningothelial cells with no significant necrosis or mitotic activity.** Hematoxylin and eosin stain; magnification, ×400.

A pathological examination revealed that the tumor tissue was composed of meningothelial cells in sheets and rudimentary whorls (Figures 4 and 5). The neoplastic cells exhibited round to oval nuclei, inconspicuous nucleoli, fine chromatin and indistinct cytoplasmic borders (Figure 6). Mitoses were seen 1 per high-power field but no necrosis was noted. Cytological atypia was noted focally and hyalinized vessels were observed. There was no evidence of malignancy. The tumor cells showed positivity for ‘vimentin’ and ‘epithelial membrane antigen’ and staining for S100 was negative. The histopathological diagnosis was ‘meningioma, World Health Organization Grade 1’.

## Discussion

A different classification of cystic meningioma has been used to define the site of cystic cavity in relation to tumoral lesion. According to Nauta *et al*. [4] cystic meningiomas are mainly divided into four categories according to the site of the cystic cavity:

1. Centrally located intratumoral cysts

2. Peripherally located intratumoral cysts

3. Peritumoral cysts in the adjacent parenchyma and

4. Peritumoral cysts between the tumor and the adjacent parenchyma

Various pathophysiological mechanisms of cyst formation in meningiomas have been postulated. Peritumoral cyst and intratumoral cyst formation have different pathophysiological mechanisms. Peritumoral cysts may be caused by reactive gliosis, fibroblastic proliferation, the final stage of the intense peritumoral edema, a widening of the subarachnoid space, or mechanical trapping of the cerebrospinal fluid spaces [5]. Conversely, intratumoral cysts are the result of microcystic degeneration, ischemic necrosis, or hemorrhage within the tumor [2]. Malignant meningiomas have a higher incidence of cyst formation due to ischemic necrosis. Pathologically, intratumoral cysts are more common in the angiomatous and meningothelial variants as opposed to peritumoral cysts which are more common in the meningothelial variant [5].

In solid meningiomas the sensitivity of a CT brain scan is virtually 100% and the specificity is 90%, however, with cystic meningiomas, the diagnosis is made pre-operatively in less than 38% of cases [2]. The presence of an enhancing nodule within a large cystic mass is a differential diagnosis along with other more common intracranial lesions like pilocytic astrocytoma and hemangioblastoma, especially in young adults, in which the appearances are similar. The commonest location of cystic meningiomas is in the cerebral convexity [2,5] particularly in the frontal and parietal regions. The cerebral falx is the second most frequent location [2]. In this case, the location of the lesion in the posterior cranial fossa and adjacent to the 4th ventricle, makes it difficult to determine radiologically whether the lesion is extra-axial or intra-axial.

Classically the MR imaging characteristics of a cystic meningioma have been described as an extra-axial lesion with enhancement of solid component and presence of a dural tail [2], which is thought to be due to increased vascularity and venous congestion within the adjacent meninges. In this case, the post-processed multiplanar MRI views showed that the lesion appeared to lie adjacent to the posterior falx, thus suggesting the possibility of an extradural origin. However, it did not exhibit any dural or cyst wall enhancement. Because a hemangioblastoma commonly presents as a well-circumscribed tumor with a solid mural nodule within a large cystic cavity [6], the difficulty of a radiological diagnosis arose. Furthermore, hemangioblastomas are typically found in the cerebellum in 83% to 86% of cases [6] whereas cystic meningiomas are commonly found at the cerebral convexities, particularly at the frontoparietal regions. Odake [7] describes three cases of cystic meningioma investigated by MRI and stated that “cystic meningiomas may not be differentiated from partly enhanced glioma or metastasis on MRI because of their non-enhanced cyst and focal oedema”.

An MRS of the enhancing solid component in this cystic meningioma did not help in differentiating between cystic meningioma and hemangioblastoma as both tumors showed an almost similar reduced N-acetylaspartate peak with increased choline-to-creatine ratio. It has been reported that the presence of high mobile lipid on proton MRS is more suggestive of hemangioblastoma, which was not seen in this case. However, the lipid peak on the MRS is non-specific and is also observed in other high-grade tumors such as high-grade gliomas, metastatic tumor, and anaplastic meningioma [8].

## Conclusion

In benign cystic meningiomas pertaining to the infratentorial variety, radiological findings utilizing the various MRI sequences and MRS and intraoperative impressions may not be reflective or in keeping with the final histopathological diagnosis.

## Consent

Written informed consent was obtained from our patient for the publication of this case report and any accompanying images. A copy of the written consent is available for review by the Editor-in-Chief of this journal.

## Competing interests

The authors declare that they have no competing interests.

## Authors’ contributions

TKG was the major contributor in writing the manuscript. DP analyzed and interpreted the patient’s data. KHT performed the histological slide preparation and interpretation. GK reviewed and interpreted the biomedical imaging results. HC and DG are the senior authors who performed the surgery, contributed and supervised the writing of the manuscript. All authors read and approved the final manuscript.
